# Dietary Green Leafy Vegetables and Cardiovascular Disease Risk: A Systematic Review and Meta-Analysis

**DOI:** 10.3390/foods15152678

**Published:** 2026-07-29

**Authors:** Thanaporn Kaewpradup, Charoonsri Chusak, Praew Chantarasinlapin, Saisha Shetty, Van Nguyen, Zhongyang Guan, Ranil Coorey, Blossom C. M. Stephan, Sirichai Adisakwattana, Mario Siervo

**Affiliations:** 1 Center of Excellence in Phytochemical and Functional Food for Clinical Nutrition, Department of Nutrition and Dietetics, Faculty of Allied Health Science, Chulalongkorn University, Bangkok 10330, Thailand; 6378301637@student.chula.ac.th (T.K.); charoonsri.c@chula.ac.th (C.C.); praew.c@chula.ac.th (P.C.); 2 Curtin-Chulalongkorn Collaborative Centre for Nutrition and Food Research and Education, Curtin University, Perth, WA 6845, Australia; r.coorey@curtin.edu.au (R.C.); blossom.stephan@curtin.edu.au (B.C.M.S.); 3 Faculty of Health Sciences, School of Population Health, Curtin University, Perth, WA 6102, Australia; saishashettyaus@outlook.com (S.S.); van.h.nguyen@curtin.edu.au (V.N.); zhongyang.guan@curtin.edu.au (Z.G.); 4 School of Molecular and Life Sciences, Curtin University, Perth, WA 6102, Australia; 5 Dementia Centre of Excellence, Enable Institute, Curtin University, Perth, WA 6102, Australia; 6 Curtin Medical Research Institute (CMRI), Curtin University, Perth, WA 6102, Australia

**Keywords:** green leafy vegetables, cardiovascular diseases, hypertension, blood pressure, vascular function, meta-analysis

## Abstract

Background: Epidemiological evidence suggests that green leafy vegetables (GLV) may reduce the risk of cardiovascular disease (CVD). However, the relationship remains incompletely understood due to inconsistencies between findings from observational studies and randomized controlled trials (RCTs). Therefore, a comprehensive synthesis of evidence across study designs is warranted. This systematic review and meta-analysis aimed to evaluate the association between GLV consumption and CVD risk, as well as related cardiovascular risk factors, including blood pressure (BP) and endothelial function, based on evidence from observational studies and RCTs. Methods: Systematic searches of PubMed, EMBASE, APA PsycInfo, and Ovid databases were conducted through 30 October 2024. Risk of bias was assessed using the ROBINS-E tool for observational studies and the RoB 2 tool for RCTs. Pooled relative risks (RRs) with 95% confidence intervals (CIs) were calculated for observational studies, whereas mean differences (MDs) were used for RCT outcomes. Random-effects meta-analyses were performed, and heterogeneity was evaluated using Cochran’s Q and I^2^ statistics. Results: Higher GLV consumption was significantly associated with a reduced risk of CVD in observational studies (RR: 0.89; 95% CI: 0.82–0.97; *p* < 0.01; I^2^ = 80%; Q = 55.88) and lower CVD mortality (RR: 0.79; 95% CI: 0.62–0.97; *p* < 0.01; I^2^ = 80%; Q = 4.93). However, pooled analysis of seven RCTs showed no significant improvement in vascular function following GLV intake (MD: −0.37; 95% CI: −1.90 to 1.16; *p* = 0.64; I^2^ = 88%; Q = 18.59). No significant associations were observed between GLV consumption and hypertension risk in observational studies, nor were significant effects found on systolic or diastolic BP in RCTs. Conclusions: Higher GLV consumption may be associated with a lower risk of CVD in observational studies. However, evidence from RCTs remains limited and inconclusive. Further well-designed clinical trials are needed to confirm these associations, establish dose–response relationships, and elucidate the mechanisms underlying the cardioprotective effects of GLVs to support evidence-based dietary recommendations.

## 1. Introduction

Cardiovascular disease (CVD) remains the leading cause of mortality and disability worldwide, with global cardiovascular deaths projected to rise by 73.4% between 2025 and 2050 [1,2]. CVD is a multifactorial disease influenced by lifestyle and metabolic risk factors, including hypertension, dyslipidemia, diabetes mellitus, physical inactivity, suboptimal dietary patterns, tobacco use, excessive alcohol consumption, obesity, and chronic psychosocial stress [3]. Hypertension is one of the most important modifiable risk factors for CVD [3], as elevated blood pressure (BP) impairs endothelial integrity, leading to endothelial dysfunction and the initiation of atherosclerosis [4]. Key mechanisms involved in this process include increased reactive oxygen species (ROS) production and sub-intimal infiltration of inflammatory cells, which contribute to plaque formation [5,6]. These atherosclerotic plaques promote arterial stiffness and reduce blood flow to vital organs, thereby increasing the risk of adverse cardiovascular events [7,8,9].

Lifestyle modifications, particularly dietary interventions, are widely recognized as effective strategies for CVD prevention and management [3]. Dietary patterns rich in plant-based foods, notably the Dietary Approaches to Stop Hypertension (DASH) and the Mediterranean diet, have demonstrated substantial cardiovascular and metabolic benefits endorsed in clinical guidelines for the prevention and management of hypertension and CVD [3,10]. These diet patterns emphasize the consumption of fruits, vegetables, whole grains, legumes, and lean proteins, while limiting saturated fats, processed foods, and added sugars, thereby improving cardiovascular outcomes through mechanisms such as enhanced antioxidant capacity, reduced inflammation, and improved lipid and glucose metabolism [10,11,12]. Nevertheless, disentangling the independent contributions of individual dietary components, particularly green leafy vegetables (GLV), is essential for developing targeted dietary recommendations. However, evidence regarding the CVD effects of GLV remains inconsistent across study designs, and most prior reviews have examined overall vegetable consumption rather than the independent effects of GLV specifically, highlighting the need for a comprehensive systematic review and meta-analysis focusing specifically on GLV.

GLV, including members of the Asteraceae family (e.g., lettuce, leafy greens) and the Brassicaceae family (e.g., cabbage, broccoli), which are commonly known as cruciferous vegetables, are consistently featured in cardioprotective diets [13]. They are rich sources of dietary fiber, vitamins, minerals, phytochemicals, and particularly inorganic nitrate, which are considered relevant to cardiovascular health [14]. Inorganic nitrate is converted to nitric oxide (NO) via the nitrate–nitrite–NO pathway, promoting vasodilation, improving endothelial function, and lowering BP [15,16]. Clinical trials have reported significant reductions in systolic and diastolic BP following GLV supplementation [17,18], although other trials have found no significant effects on 24-h ambulatory BP [19,20]. In contrast, observational studies have more consistently demonstrated protective associations between GLV intake and reduced CVD risk [21,22]. Given the limited number of studies evaluating individual GLV families separately, inclusion of both botanical families allowed a more comprehensive synthesis of the currently available evidence.

This systematic review and meta-analysis aim to synthesize current evidence from both observational studies and randomized controlled trials (RCTs) examining the association between GLV consumption and cardiovascular health. Observational studies will be assessed to determine associations between GLV intake and cardiovascular risk or outcomes, while RCTs will be evaluated for their effects on specific clinical parameters, including blood pressure, arterial stiffness, endothelial function, and platelet aggregation.

## 2. Materials and Methods

This systematic review and meta-analysis is reported according to the Preferred Reporting Items for Systematic Reviews and Meta-analyses (PRISMA) 2020 guidelines [23]. The study protocol was registered in the PROSPERO International Prospective Register of Systematic Reviews (Registration No. CRD42023449227).

### 2.1. Search Strategy

A systematic literature search was conducted from database inception to 30 October 2024 across Embase, APA PsycInfo, MEDLINE, and All Journals@Ovid via the Ovid platform. The search strategy incorporated predefined keywords and controlled vocabulary, refined through a preliminary scoping search to ensure retrieval of relevant studies before consistent application across all databases. The search strategy incorporated both free-text keywords and controlled vocabulary terms related to GLV (e.g., “leafy vegetables,” “spinach,” “kale,” “broccoli,” “cruciferous vegetables”) and cardiovascular outcomes (e.g., “cardiovascular disease,” “hypertension,” “arterial stiffness,” “endothelial function,” “platelet aggregation,” “blood pressure,” “atherosclerosis”) as shown in Supplementary Materials. The search was limited to English-language publications and Full-text. In addition, manual searches of reference lists were performed to identify relevant studies that may have been missed during the electronic search. Details of the complete search strategy are provided in the Supplementary Materials.

### 2.2. Inclusion and Exclusion Criteria

Studies were selected based on predefined eligibility criteria following the PICOS criteria presented in Table 1. Eligible study designs included observational studies (case–control, cross-sectional, and cohort studies) and RCTs, without exclusions based on participants’ health status, medication use, smoking behavior, or alcohol consumption. Eligible studies met the following criteria: (1) enrolled adult participants aged ≥18 years; (2) classified or included GLV as a distinct dietary exposure or intervention. GLV exposure was operationally defined as vegetables belonging to the Asteraceae (leafy vegetables) and Brassicaceae (cruciferous vegetables) families, such as the consumption of spinach, mustard greens, collard greens, basella, broccoli, lettuce, and other leafy greens, consistent with previous nutritional epidemiology studies examining GLV intake and health outcomes [13]; and (3) included whole vegetables as well as preparations directly derived from these vegetables (e.g., juices, powders, concentrates, extracts, and isolated bioactive compounds derived from GLV), provided that the botanical source was clearly identified.

Studies that grouped GLVs with non-GLV vegetables (e.g., beetroot) without separate reporting of GLV-specific effects were excluded. Eligible studies reported at least one cardiovascular outcome. Observational studies assessed associations between GLV consumption and cardiovascular disease outcomes (CVD incidence, CVD mortality, and hypertension incidence), while RCTs evaluated effects of GLV interventions on cardiovascular risk factors (blood pressure, endothelial function, arterial stiffness, and platelet aggregation). This dual approach captured both epidemiological associations with clinical endpoints and mechanistic effects on intermediate risk factors. No restrictions were placed on the study duration.

### 2.3. Study Selection

Study selection was conducted using Covidence systematic review software (Veritas Health Innovation, Melbourne, Australia) based on the PICOS criteria shown in Table 1. Duplicate records were initially identified automatically using Covidence and subsequently verified manually by the reviewers to confirm duplicate matches before removal. Titles and abstracts were independently screened by two reviewers (T.K. and S.S.) based on the predefined inclusion criteria. Full-text articles of potentially eligible studies were then retrieved and assessed independently by the same reviewers. Each excluded study was assigned a single primary reason for exclusion in Covidence. Studies that met all eligibility criteria were included in the final review. Any disagreements during the screening or full-text review process were resolved through discussion. If consensus could not be reached, a third reviewer (M.S.) adjudicated to make the final decision.

#### Data Extraction and Quality Assessment

Data extraction was performed using a structured Excel workbook, capturing key information across several domains: study characteristics (author, country, year of publication, study design, sample size, study duration, conflict of interest), population characteristics (sex, age, BMI, health status, medication use, physical activity), GLV consumption data (type of GLV, formulation, quantity, dietary assessment tools), and the cardiovascular disease (CVD) outcome assessed. Additionally, methods used to assess GLV intake and cardiovascular outcomes were extracted. However, no overlapping study populations or duplicate datasets were identified among the included studies.

For RCTs, data extraction included baseline characteristics and outcome measures at study completion for both intervention and control groups. Two reviewers (T.K. and S.S.) independently conducted data extraction, and a third reviewer (M.S.) verified all entries to ensure accuracy. The quality of observational studies was assessed using the Risk Of Bias In Non-randomized Studies of Exposures (ROBINS-E) tool, which evaluates the bias in participant selection, exposure classification, confounding, missing data, outcome measurement, and selective reporting [24]. Studies were categorized as low, moderate (some concerns), high, and very high risk of bias. The quality of RCTs was assessed using the revised Cochrane Risk of Bias tool (RoB 2.0; The Cochrane Collaboration, London, UK), evaluating bias domains of the effect of GLV interventions on cardiovascular outcomes at the end of the intervention [25]. The evidence quality was evaluated using the GRADE approach through GRADEpro GDT software (Evidence Prime Inc., Hamilton, ON, Canada) [26].

### 2.4. Meta-Analysis

The primary outcome for the meta-analysis of observational studies was the incidence of CVD associated with GLV consumption. Secondary outcomes included CVD mortality and hypertension risk. Included studies that classified GLVs into leafy green vegetables and cruciferous vegetables, and subgroup analyses were conducted to assess their effects separately. The effect estimates, including hazard ratios, odds ratios, or relative risks, were converted to relative risks before meta-analysis, and 95% confidence intervals comparing the highest versus lowest categories of GLV consumption were extracted, prioritizing the most fully adjusted models.

For RCTs, the key outcomes included changes in systolic and diastolic blood pressure between baseline and intervention, vascular function measures including pulse wave velocity (PWV) and flow-mediated dilation (FMD), and platelet aggregation parameters following GLV interventions. Blood pressure measurements were categorized by assessment method (24-h ambulatory monitoring, resting clinic measurements, office measurements, daytime, or nighttime measurements). When studies reported multiple blood pressure measurement methods, analyses were stratified by measurement type to minimize non-independence of observations. For data extraction of RCTs with parallel and cross-over designs, baseline and post-intervention means and standard errors reported for all outcomes were extracted. When standard deviations were not reported, they were derived from standard errors, confidence intervals, or *p*-values using standard formulas. For studies reporting median and interquartile range, means and standard deviations were estimated using established conversion methods.

A random effects model was performed using restricted maximum likelihood (REML) estimation to account for between-study heterogeneity arising from differences in study design and outcome measures. Forest plots were generated to summarize the overall associations and effects of GLV consumption according to outcome hierarchy. Primary outcomes included CVD incidence from observational studies. Secondary outcomes encompassed CVD mortality and hypertension incidence from observational studies, and changes in systolic and diastolic blood pressure, flow-mediated dilation (FMD), pulse wave velocity (PWV), and platelet aggregation from RCTs. Results were reported as relative risk (RR) with 95% confidence intervals (CI) for observational studies. For RCTs, mean differences (MD) with 95% CI were reported for each cardiovascular outcome. A sensitivity analysis was conducted to assess the impact of studies classified as very high risk of bias by the RoB2 assessment on the overall interpretation of findings from observational studies and randomized controlled trials (RCTs). Statistical heterogeneity across studies was assessed using the I^2^ statistic and Cochran’s Q test. I^2^ values of 25, 50, and 75 percent were interpreted as indicating low, moderate, and high heterogeneity, respectively. A *p* value less than 0.10 was considered statistically significant for heterogeneity [27]. Funnel plots and Egger’s regression test were used to assess potential publication bias, with a *p* value less than 0.05 indicating statistical significance. All meta-analyses were conducted using IBM SPSS Statistics software (version 29.0; IBM Corporation, Armonk, NY, USA).

## 3. Results

### 3.1. Literature Search

**Figure 1:**
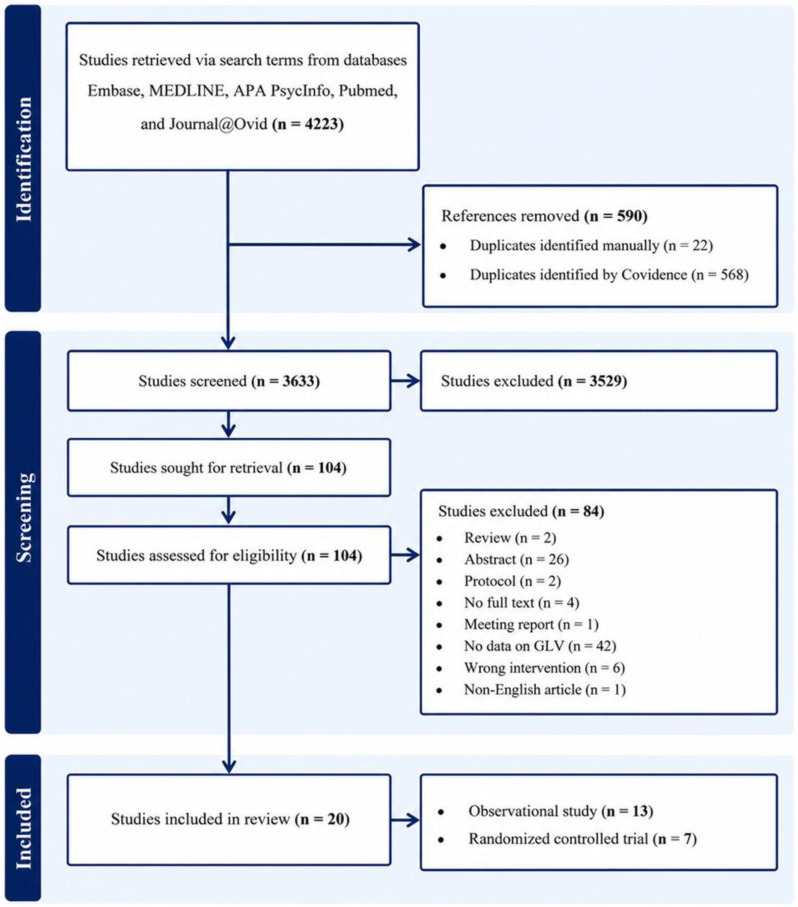
The PRISMA flow diagram describes study selection.

The selection of eligible articles is summarized in Figure 1. The initial literature search identified 4223 records, of which 3633 remained after the removal of duplicates for title and abstract screening. Based on this screening, 104 studies were selected for full-text review. Among these, 84 studies were excluded due to one or more of the following reasons: unavailability of full text (e.g., conference abstracts), absence of data related to green leafy vegetables (GLV), wrong intervention, non-English language, or non-original research (e.g., reviews, protocols, meeting reports). Ultimately, 20 studies met all inclusion criteria and were included in the review, comprising 13 observational studies and 7 RCTs.

### 3.2. Study Characteristics

#### 3.2.1. Observational Studies

Of the 13 observational studies included, 11 employed a cohort design [22,28,29,30,31,32,33,34,35,36,37], one was cross-sectional [38], and one had a case–control design [39]. The total number of participants was 717,782, with 520,822 women and 196,959 men. The total sample size across all studies was 717,782 participants, comprising 520,822 women and 196,959 men. The mean age ranged from 42.5 to 72.1 years, and the mean body mass index (BMI) ranged from 23.9 to 27.0 kg/m^2^, although BMI was not reported in three studies [28,32,39]. Four studies included only female participants [28,29,34,35], while the remaining nine studies enrolled both male and female participants [22,30,31,32,33,36,37,38,39]. The mean follow-up duration was 14.2 years, ranging from 4 to 24 years. Green leafy vegetable (GLV) intake was classified into two categories: general GLV and cruciferous vegetables. To assess GLV consumption, two studies used a standard food frequency questionnaire (FFQ), whereas eleven studies employed a semi-quantitative FFQ. Cardiovascular outcomes (CVD risk, CVD mortality, and hypertension risk) were assessed using various methods, including medical records, in-person interviews, self-reported data, and hospital-based diagnoses (Table 2).

#### 3.2.2. Randomized Clinical Trials

Seven RCTs were included in the meta-analysis [19,20,40,41,42,43,44] (Table 3). These consisted of four randomized controlled crossover trials [20,41,42,43], two parallel RCTs [40], and one crossover trial that did not clearly report randomization procedures [44]. The total number of participants across all studies was 396, with individual study sample sizes ranging from 12 to 122. The average age of participants was 54.7 years (range: 29.5 to 68.0 years), and the average body mass index (BMI) was 26.3 kg/m^2^ (range: 23.6 to 28.1 kg/m^2^); however, BMI data were not reported in one study [44]. Six studies included both male and female participants [19,20,40,41,42,43], while one study was conducted exclusively in females [44]. With respect to participants’ health status, five studies enrolled individuals at high risk of CVD [19,20,40,43,44], and two were conducted in healthy adults [41,42]. The average duration of the intervention was 13 days, ranging from 1 to 35 days. The types of GLV investigated included leafy greens, cruciferous vegetables, or both, with some studies evaluating specific varieties such as lettuce, broccoli, or cauliflower in various forms, including raw vegetables and extracts. All seven RCTs assessed changes in blood pressure, including both systolic blood pressure (SBP) and diastolic blood pressure (DBP), while three studies additionally assessed endothelial function [19,40,42] and arterial stiffness [20,41,43].

### 3.3. Meta-Analysis

#### 3.3.1. Observational Studies

##### CVD Risk

**Figure 2:**
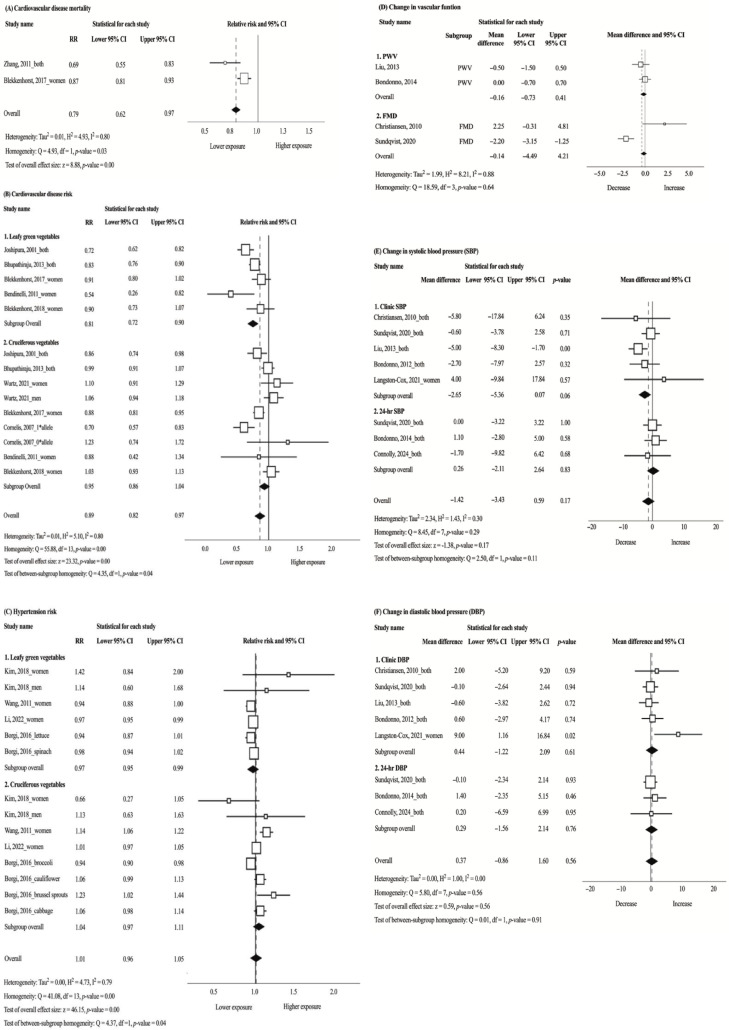
Forest plots illustrate the association between green leafy vegetable (GLV) consumption and cardiovascular disease (CVD) incidence and related outcomes [19,20,22,28,29,30,31,32,33,34,35,36,37,38,39,40,41,42,43,44]. Pooled effect estimates were calculated using random-effects meta-analysis. Effect estimates for observational studies are presented as relative risks (RRs) with 95% confidence intervals (CIs), while estimates for randomized controlled trials (RCTs) are presented as mean differences (MDs) with 95% CIs. Heterogeneity statistics are displayed within each forest plot. (**A**) shows cardiovascular disease mortality; (**B**) shows CVD risk; (**C**) presents hypertension risk; (**D**) displays MD in vascular function [Pulse Wave Velocity (PWV, m/s) and Flow Mediated Dilation (FMD, %)]; (**E**) presents MD in systolic blood pressure (mmHg); and (**F**) shows MD in diastolic blood pressure (mmHg).

Higher consumption of all GLV types was associated with a 11% reduction in overall CVD risk (RR: 0.89; 95% CI: 0.82, 0.97; 95% prediction interval (95% PI): 0.72, 1.10; Figure 2B). Subgroup analysis showed a similar association for leafy green vegetables (RR: 0.81; 95% CI: 0.72, 0.90), whereas there was no association with cruciferous vegetable consumption (RR: 0.95; 95% CI: 0.86, 1.04). Additionally, higher GLV consumption was associated with a 21% reduction in CVD mortality (RR: 0.79; 95% CI: 0.62, 0.97; 95% PI: 0.59–1.06; Figure 2A). Funnel plot asymmetry and Egger’s regression test indicated potential publication bias for CVD risk outcomes (Supplementary Material Figure S1 and Table S4). The certainty of the evidence was rated as moderate for CVD risk and mortality, downgraded for risk of bias and inconsistency (Supplementary Material Table S3). The exclusion of a study with a high risk of bias [32] did not modify the overall results.

##### Hypertension Risk

Hypertension risk: Overall, green leafy vegetable (GLV) consumption was not associated with hypertension risk (RR: 1.01; 95% CI: 0.96, 1.05; 95% PI: 0.97, 1.06; Figure 2C). However, stratified analysis by GLV type revealed that a higher intake of leafy green vegetables was associated with a modest reduction in hypertension risk (RR: 0.97; 95% CI: 0.95, 0.99), whereas no association was observed for cruciferous vegetable consumption (RR: 1.04; 95% CI: 0.97, 1.11; Figure 2C). In addition, funnel plot asymmetry was observed, and Egger’s regression test confirmed the presence of publication bias (Supplementary Material Figure S1 and Table S4). The certainty of evidence was rated as moderate for hypertension risk, downgraded for inconsistency and indirectness (Supplementary Material Table S3). The exclusion of a study with high risk of bias [33] did not modify the overall results.

**Table 2:** Characteristics of observational studies included in the systematic review and meta-analysis.

Study	Country	Study Design	Population	Sample Size (N)	Women (N)	Age ^a^ (years)	BMI ^a^ (kg/m^2^)	Follow-Up (Years)	GLV Type	Dietary Intake Assessment	Lower Intake	Higher Intake	Outcome	Outcome measurement
Joshipura et al., 2001 [32]	United States	Cohort study	NHS and HPFS	126,399	84,251	Women: 34–59 Men: 40–75	Not reported	Women: 14 Men: 8	LF, CF	61-item semi-FFQ	0.14 and 0.16 servings/d for CF and LF in both women and men	0.95 and 1.01 servings/d for CF in women and men 1.51 and 1.36 servings/d for LF in women and men	CVD risk	Medical record
Bhupathiraju et al., 2013 [22]	United States	Cohort study	NHS and HPFS	113,276	71,141	Women: 30–55 Men: 40–75	Women: 25 Men: 25	Women: 24 Men: 22	LF, CF	126-item semi-FFQ	0.14 servings/d for CF in both women and men, while 0.22 and 0.14 servings/d for LF in women and men	0.92 and 1.00 servings/d for CF in women and men 1.50 and 1.43 servings/d for LF in women and men	CVD risk	Medical record
Zhang et al., 2011 [37]	China	Cohort study	SWHS and SMHS	134,796	73,360	Women: 40–70 Men: 40–74	Women: 24 Men: 23.7	Women: 10.2 Men: 4.6	CF	FFQ	28 and 34 g/d in women and men	166 and 208 g/d in women and men	CVD mortality	In-person interview and medical record
Ascherio et al., 1996 [28]	United States	Cohort study	NHS	41,541	41,541	42.5	Not reported	4	LF	61-item semi-FFQ and 126-item semi-FFQ	Not reported	Not reported	Hypertension risk	Self-reported and medical records
Kim et al., 2018 [33]	Korea	Cohort study	KoGES	4257	2172	50.5	24.2	9	LF, CF	103-item semi-FFQ	<3–4 servings/wk	≥1 servings/d	Hypertension risk	Medical records
Wang et al., 2011 [35]	United States	Cohort study	WHS	28,082	28,082	54	25.2	12.9	LF, CF	131-item semi FFQ	<0.2 servings/d	≥1 servings/d	Hypertension risk	Self-reported and medical records
Li et al., 2022 [34]	China	Cohort study	NHS	55,233	55,233	54	24.7	12	LF, CF	116-item semi-FFQ and 126-item semi-FFQ	0.3 serving/d for both LF and CF	1.5 and 0.7 serving/d for LF and CF	Hypertension risk	Self-reported and medical records
Wurtz et al., 2021 [36]	Denmark	Cohort study	Danish Diet, Cancer and Health cohort	55,171	29,142	Women: 56 Men: 56	Women: 25.5 Men: 26.5	13.6	CF	192-item semi-FFQ	Substitution with 200 g/week	None	CVD risk	Hospital diagnosis and medical records
Blekkenhorst et al., 2017 [30]	Australia	Cohort study	PLSAW	1227	1226	75	27	15	LF, CF	Semi-FFQ	13.3 ± 9.4 and 18.9 ± 12.8 g/d for LF and CF	23.4 ± 13.4 and 46.0 ± 25.1 g/d for LF and CF	CVD mortality	Medical records and FFQ
Blekkenhorst et al., 2018 [38]	Australia	Cross-sectional study	CAIFOS	968	968	75	26.9	4	LF, CF	Semi-FFQ	13.0 ± 9.2 and 18.6 ± 12.8 g/d for LF and CF	23.3 ± 13.6 and 46.0 ± 24.8 g/d for LF and CF	CVD risk	Clinical assessment and medical records
Cornelis et al., 2007 [39]	Canada	Case–control study	Costa Rica’s Central Valley	4084	968	58	NA	11	CF	135-item semi-FFQ	0.08 servings/d	0.86 servings/d	CVD risk	Hospital-based clinical diagnosis
Borgi et al., 2016 [31]	United States	Cohort study	NHS, HNS II and HPFS	123,059	103,049	46	24.4	8	LF, CF	FFQ	<1 per month of consumption level	≥4 per week of consumption level	Hypertension risk	Self-reported
Bendinelli et al., 2011 [29]	Italy	Cohort study	EPIC	29,689	29,689	50	25.6	7.9	LF, CF	The EPIC-Italy FFQ	≤17.6 and ≤1.2 g/d for LF and CF	>50.8 and >8.2 g/d for LF and CF	CVD risk	Medical records

^a^ Values are presented as range or means; N, number of participants; CVD, Cardiovascular disease; NHS, The Nurses’ Health Study; HPFS, The Health Professionals Follow-Up Study; SWHS, The Shanghai Women’s Health Study; SMHS, The Shanghai Men’s Health Study; KoGES, The Korean Genome and Epidemiology Study; WHS, The Women’s Health Study; PLSAW, The Perth Longitudinal Study of Ageing Women; CAIFOS, the Calcium Intake Fracture Outcome Study; EPIC, The European Prospective Investigation into Cancer and Nutrition study; LF, Leafy green vegetables; CF, Cruciferous vegetables; FFQ, Food Frequency Questionnaire; NA, Not applicable.

**Table 3:** Characteristics of the randomized controlled trials included in the systematic review and meta-analysis.

Study	Country	Study Design	Sample Size (N)	Women (N)	Health Status	Age ^a^ (Years)	BMI ^a^ (kg/m^2^)	Study Duration (Days)	GLV Type	Type of Intervention	Type of Control	Outcome
Christiansen et al., 2010 [40]	Denmark	Parallel randomized controlled trial	41	25	High CVD risk	56	27.7	28	CF	10 g of dried sprouts (100 g of fresh sprouts)	10 g of dried sprouts (freezing/thawing before drying)	Change in blood pressure and endothelial function
Sundqvist et al., 2020 [19]	Sweden	Parallel randomized controlled trial	231	122	High CVD risk	63	25.8	35	LF, CF	150 mg nitrate from leafy green vegetables, twice daily	150 mg nitrate from potassium nitrate, twice daily	Change in blood pressure and endothelial function
Liu et al., 2013 [41]	Australia	Randomized controlled cross-over study	26	20	Healthy	59	25.4	1	LF	White bread and comprised chicken and cheese with 250 g of cooked spinach (200 g nitrate)	White bread and comprised chicken and cheese without 250 g of cooked spinach	Change in blood pressure and arterial stiffness
Bondonno et al., 2014 [20]	Australia	Randomized controlled cross-over study	38	26	High CVD risk	61	27.1	7	CF	High nitrate diet (nitrate intake at least 300 mg/day from green leafy vegetables)	Low nitrate diet (nitrate intake less than 100 mg/day)	Change in blood pressure and arterial stiffness
Bondonno et al., 2012 [42]	Australia	Randomized controlled cross-over study	30	24	Healthy	47	23.6	1	LF	Spinach active consisted of 200 g of spinach (204 kJ)	Low-flavonoid apple control and low-nitrate control consisted of 200 g of apple flesh	Change in blood pressure and endothelial function
Connolly et al., 2024 [43]	Australia	Randomized controlled cross-over study	18	16	High CVD risk	68	28.1	14	CF	4 servings of vegetable soup containing GLV vegetables (approximately 300 g/day)	4 servings of vegetable soup containing root vegetables (approximately 300 g/day)	Change in blood pressure and arterial stiffness
Langston-Cox et al., 2021 [44]	Australia	Crossover trial	12	12	High CVD risk	30	Not reported	1	CF	8 capsules of broccoli seed extract (64 mg sulforaphane)	4 capsules of broccoli seed extract (32 mg sulforaphane)	Change in blood pressure

^a^ Values are presented as means; N, number of participants; CVD, Cardiovascular disease; LF, Leafy green vegetables; CF, Cruciferous vegetables.

#### 3.3.2. Randomized Clinical Trials

##### Effect of GLV Consumption on Vascular Function

Green leafy vegetable (GLV) consumption was not associated with improvements รin arterial stiffness, assessed by PWV (MD: −0.16; 95% CI: −0.73, 0.41) or endothelial function, evaluated by FMD (MD: −0.14; 95% CI: −4.49, 4.21) (Figure 2D). The 95% PI for overall vascular function (−3.53 to 2.79) indicated considerable between-study variability. No evidence of publication bias was detected by funnel plot analysis or Egger’s regression test (Supplementary Material Figure S2 and Table S4). The certainty of evidence was rated as moderate, downgraded for imprecision (Supplementary Material Table S3).

##### Effect of GLV Consumption on Blood Pressure

GLV consumption was not associated with a significant reduction in SBP compared to control groups (MD: −1.42; 95% CI: −3.43, 0.59; Figure 2E). Subgroup analysis showed no significant reduction in either resting clinic SBP (MD: −2.65; 95% CI: −5.36, 0.07) or 24-h SBP (MD: 0.26; 95% CI: −2.11, 2.64; Figure 2E). Similarly, GLV consumption had no significant effect on DBP overall (MD: 0.37; 95% CI: −0.86, 1.60), or in subgroup analyses by measurement type, including resting clinic DBP (MD: 0.44; 95% CI: −1.22, 2.09) and 24-h ambulatory DBP (MD: 0.29; 95% CI: −1.56, 2.14; Figure 2F). No significant publication bias was detected for either SBP or DBP as assessed by funnel plot analysis and Egger’s regression test (Supplementary Material Figure S2 and Table S4). The certainty of the evidence was rated as low for both systolic and diastolic blood pressure, downgraded for risk of bias (Supplementary Material Table S3).

### 3.4. Quality of Study

Most observational studies were rated as low risk of bias based on the ROBINS-E tool assessment [28,29,30,31,34,35,36,37,38,39], whereas one study was rated as some concern for risk of bias due to post-exposure intervention [22] with the other two studies assessed as high risk of bias due to insufficient detail regarding the selection of the reported result [32,33]. One study was excluded from the meta-analysis due to the absence of outcome data relevant to the analysis [28]. Overall, 10 out of 13 observational studies (77%) were considered to have a low risk of bias. Among the RCTs, six out of seven studies (86%) were classified as low risk of bias [19,20,40,41,42,43,44], whereas one study [44] was rated as high risk of bias due to unclear randomization and lack of blinding procedures according to the RoB 2.0 assessment. Detailed results of the quality assessments are presented in Supplementary Material Tables S1 and S2.

## 4. Discussion

This systematic review and meta-analysis synthesized evidence from observational studies and RCTs to evaluate the association between GLV consumption and cardiovascular health. Higher GLV consumption was associated with reduced CVD incidence and mortality in observational studies. However, evidence from RCTs did not demonstrate consistent improvements in blood pressure or vascular function. These findings underscore the importance of distinguishing long-term epidemiological associations from short-term intervention effects and extend current dietary recommendations [45,46,47] by suggesting that GLVs may represent a distinct group of vegetables with potential cardiovascular benefits that deserve further investigation.

Findings from large prospective cohort studies in this review revealed that higher GLV consumption was associated with a 12% reduction in CVD risk and a 21% reduction in CVD mortality. These findings are consistent with a previous meta-analysis of 17 prospective studies reporting a 7% reduction in total CVD events associated with greater vegetable intake [48]. Additional studies further support these results; one found 13% and 15% reductions in CVD mortality with higher intake of leafy green and cruciferous vegetables, respectively [49], while another reported 22% and 10% reductions, respectively, for the same vegetable types [50]. These protective effects may be attributed to bioactive compounds in GLV, such as inorganic nitrate, vitamin C, potassium, and a variety of phytonutrients, which influence cardiovascular mechanisms, including blood pressure regulation and endothelial function [51].

Notably, the nitrate–nitrite–nitric oxide (NO) pathway is a key mechanism through which inorganic nitrate in GLVs contributes to vasodilation and endothelial protection [52,53,54]. Additionally, potassium promotes sodium excretion and reduces fluid retention, which collectively help regulate BP and reduce hypertension and CVD risk [55,56].

Although the included studies varied in GLV subtype, intervention formulation, participant characteristics, and follow-up duration, statistical pooling was deemed appropriate due to interventions that evaluated GLV or preparations directly derived from GLV and addressed common cardiovascular outcomes. A random-effects model was employed to account for anticipated clinical and methodological heterogeneity. Nonetheless, these sources of between-study variability should be considered when interpreting the pooled estimates. However, the prediction interval crossed the null value, suggesting that the magnitude and direction of the association may vary across different populations and settings.

While no overall association between total GLV consumption and hypertension risk was observed, leafy green vegetables alone were associated with a modest risk reduction. This finding aligns with prior meta-analyses demonstrating heterogeneous cardiovascular effects across vegetable subtypes. For instance, some GLVs such as lettuce and broccoli have been inversely associated with CVD risk, whereas evidence for other GLV subtypes has been inconsistent or inconclusive [57]. Such variability likely reflects differences in phytochemical profiles among GLV subtypes, contributing to differential physiological effects [58]. The null finding may reflect incomplete dietary confounder control, as most studies adjusted only for total fruit and vegetable intake rather than specific food groups. Residual confounding remains a concern, as dietary intake was primarily assessed via FFQs or other self-reported methods, which are susceptible to recall bias and measurement error, potentially attenuating the observed associations despite adjustment for major confounders. Additionally, GLVs are typically consumed as part of broader dietary patterns, limiting the ability to isolate their independent cardiovascular effects.

These observational findings are consistent with RCTs in this review, which reported no significant reductions in systolic or diastolic BP following GLV interventions. Subgroup analysis showed that only clinic-measured SBP showed marginal improvement, while clinic DBP and 24-h ambulatory BP showed no change. These mixed results may reflect methodological variability in BP assessment and the inability to evaluate specific GLV subtypes due to limited sample sizes.

Although no prior meta-analysis has specifically assessed GLVs in relation to BP, our findings align with broader research on vegetable consumption. For example, a meta-analysis involving individuals classified as having overweight or obesity reported modest reductions in SBP but no changes in DBP with increased fruit and vegetable intake [59]. These inconsistencies may be due to differences in GLV types, bioactive compound concentrations, or dose–response relationships across studies [60].

No significant associations were observed between GLV consumption and vascular function parameters in our meta-analysis of RCTs, despite an overall modest improvement compared to control groups. The included RCTs varied in sample size, ranging from 12 to 231 participants, contributing to high variability in effect sizes. Several factors may explain the lack of statistical significance and the substantial heterogeneity observed across the included RCTs. Although all interventions were derived from GLV, the studies differed considerably in the type of vegetables investigated, with most trials evaluating either leafy green vegetables or cruciferous vegetables individually and only one study including both. In addition, there was substantial variation in intervention formulation (whole vegetables, powders, or extracts), dosage, intervention duration, and participant characteristics. Differences in dosage were also considerable, which may have influenced the concentration of cardiovascular bioactive compounds delivered in each study [60]. These methodological and clinical differences may have influenced the delivery and bioavailability of bioactive compounds, contributing to heterogeneous physiological responses and variability in the pooled estimates.

Moreover, intervention duration ranged from 1 to 35 days, and shorter durations may not have been sufficient to elicit meaningful changes in vascular function. The timing of phytonutrient intake plays a critical role in modulating postprandial oxidative stress and vascular processes [61,62]. This interpretation is supported by findings from a study in our analysis, which reported that consuming a high nitrate GLV diet for 7 days did not effectively improve arterial stiffness [20]. Our findings are also consistent with a previous systematic review of overall vegetable intake, which similarly reported no significant improvements in FMD and PWV, despite including fewer studies and a broader range of vegetable types [63]. That review also highlighted similar issues with heterogeneity and methodological variation.

Although our meta-analysis included a greater number of studies specifically focused on GLV, the persistent absence of statistically significant effects suggests that current evidence is insufficient to confirm definitive vascular benefits. Future research should employ more standardized protocols, longer intervention durations, and more homogeneous study populations to better elucidate the vascular effects of GLV consumption.

The study has notable strengths. First, the systematic review provides a comprehensive classification of cardiovascular outcomes by assessing the association between GLV consumption and multiple cardiovascular parameters, including CVD risk, hypertension risk, BP changes, and vascular function. Notably, the analysis included both clinic BP and 24-h ambulatory BP, as well as vascular function measures such as FMD and PWV, offering physiological insights into underlying mechanisms. Second, we distinguished between specific GLV subgroups, namely leafy green vegetables and cruciferous vegetables, rather than treating them as a homogeneous category. This differentiation acknowledges their distinct nutritional compositions and bioactive compounds, allowing for a more nuanced interpretation of their potential cardiovascular benefits.

Third, the inclusion of studies across diverse populations, including individuals with CVD, hypertension, and diabetes, enhances the external validity and generalizability of the findings across varied health statuses. Collectively, this multifaceted approach offers robust evidence supporting GLV consumption as a promising dietary strategy for cardiovascular disease prevention, while also shedding light on multiple pathophysiological pathways involved.

Despite its strengths, this review has several limitations. A dose–response meta-regression was not performed because the limited number of eligible studies and inconsistent reporting of dietary intake precluded harmonization of exposure levels across studies. Furthermore, substantial variation in vegetable subtype, intervention formulation, participant characteristics, intervention duration, consumption quantity, and small sample sizes may reduce statistical power and the precision of effect estimates, contributing to heterogeneity and limiting the interpretation of the pooled findings. Only a limited number of studies examined platelet aggregation, representing a significant knowledge gap in understanding the mechanistic role of GLV intake in cardiovascular health. Several methodological concerns were also noted. Some randomized controlled trials (RCTs) lacked clear descriptions of blinding and randomization procedures, raising concerns of potential bias. Additionally, the duration of interventions varied widely, ranging from acute to long-term studies, limiting our ability to distinguish temporal patterns of GLV effects. In the case of observational studies, there was substantial heterogeneity in GLV exposure assessment, including differences in food frequency questionnaires (FFQs), dietary recall periods, and GLV categorization methods. Moreover, the reliance on self-reported dietary data introduces additional limitations, including recall bias, social desirability bias, and systematic under-reporting or over-reporting of food intake. These measurement errors may have resulted in exposure misclassification, potentially attenuating observed associations and contributing to heterogeneity across studies. Further limitations include variability in participants’ baseline health conditions and follow-up durations, both of which may influence cardiovascular outcomes. Individuals with existing chronic conditions may have already altered their diets, potentially introducing reverse causality or confounding. Shorter follow-up periods may also limit the detection of long-term cardiovascular benefits. Finally, although the review focused on key cardiovascular markers such as BP, vascular function, and platelet aggregation, other relevant cardiovascular outcomes (e.g., lipid profiles, inflammatory markers, or clinical events) were not included, leaving certain dimensions of GLV’s cardiovascular impact unexplored.

## 5. Conclusions

This systematic review and meta-analysis suggest that higher GLV consumption was associated with a lower risk of cardiovascular disease and cardiovascular mortality in observational studies. However, evidence from randomized controlled trials remains limited and has not consistently demonstrated beneficial effects on blood pressure or vascular function. Together, these findings support an association between GLV consumption and cardiovascular health but do not establish causality. Further well-designed, long-term randomized controlled trials are needed to clarify dose–response relationships, identify the most beneficial GLV subtypes, and improve understanding of the biological mechanisms underlying the cardiovascular effects of GLV.

## Figures and Tables

**Table 1:** The PICOS criteria for inclusion of studies.

Parameters	Criteria
Population (P)	Adult participants aged ≥18 of both sexes with or without limitation on health status.
Intervention or exposure (I/E)	All varieties of GLV consumption (e.g., spinach, broccoli, lettuce, mustard/collard greens, basella) delivered in any form, including whole food, juice/beverage, powder extract, or isolated bioactive compounds, aimed at reducing CVD incidence or prevalence or hypertension.
Comparison (C)	OR, RR, or HR for CVD incidence/mortality (high vs. low GLV intake), and MD CVD-related risk factors.
Outcome (O)	Change in cardiovascular risk and CVD-related risk factors (including blood pressure, endothelial function, arterial stiffness, and platelet aggregation.
Types of study (S)	A randomized clinical trial and observational studies examining the effects of green leafy vegetable consumption on hypertension and cardiovascular health

## Data Availability

No new data were created or analyzed in this study. Data sharing is not applicable to this article.

## Supplementary Materials

The following supporting information can be downloaded at: https://www.mdpi.com/article/10.3390/foods15152678/s1, Figure S1: Funnel plot indicating the presence of publication bias in cardiovascular disease incidence; Figure S2: Funnel plot indicating the presence of publication bias in vascular function and blood pressure change; Table S1: Risk of bias of observational studies included in the systematic review and meta-analysis using the ROBINS-E assessment tool; Table S2: Risk of bias of randomized controlled trials included in the systematic review and meta-analysis using the RoB2 assessment tool; Table S3: GRADE Analysis; Table S4: The Egger’s regression test for publication bias.

## Abbreviations

The following abbreviations are used in this manuscript:

GLV	Green leafy vegetable
RCTs	Randomized controlled trial
CVD	Cardiovascular disease
BP	Blood pressure
SBP	Systolic blood pressure
DBP	Diastolic blood pressure
Semi-FFQ	Semi-quantitative food frequency questionnaire
PWV	Pulse wave velocity
FMD	Flow-mediated dilation
RR	Relative risk
MD	Mean differences
95% CI	95% confidence intervals
