# Supplementary material for: Dietary Green Leafy Vegetables and Cardiovascular Disease Risk: A Systematic Review and Meta-Analysis
Source: Foods. 2026 Jul 29;15(15):2678. doi: 10.3390/foods15152678 (PMC13465923; doi:10.3390/foods15152678)
Supplement: Supplementary file 1 [file foods-15-02678-s001.zip › foods-4381081-supplementary.pdf]

Dietary Green Leafy Vegetables and Cardiovascular Disease Risk: A Systematic Review and Meta-Analysis

Thanaporn Kaewpradup[1,2], Charoonsri Chusak[1,2], Praew Chantarasinlapin[1,2], Saisha Shetty[3], Van Nguyen[3], Zhongyang Guan[3], Ranil Coorey[2,4], Blossom CM Stephan[2,5], Sirichai Adisakwattana[1,2,* #], and Mario Siervo[2,3,5,6#]

[1] Phytochemical and Functional Food Research Unit for Clinical Nutrition, Department of Nutrition and Dietetics, Faculty of Allied Health Science, Chulalongkorn University, Bangkok, 10330, Thailand; 6378301637@student.chula.ac.th (T.K.); charoonsri.c@chula.ac.th (C.C.); praew.c@chula.ac.th (P.C.); sirichai.a@chula.ac.th (S.A.)

[2] Curtin-Chulalongkorn Collaborative Centre for Nutrition and Food Research and Education, Curtin University, Perth, WA, Australia and Chulalongkorn University, Bangkok, Thailand; 6378301637@student.chula.ac.th (T.K.); charoonsri.c@chula.ac.th (C.C.); praew.c@chula.ac.th (P.C.); Blossom.Stephan@curtin.edu.au (B.S.); mario.siervo@curtin.edu.au (M.S.); sirichai.a@chula.ac.th (S.A.); R.Coorey@curtin.edu.au (R.C.)

[3] Faculty of Health Sciences, School of Population Health, Curtin University, Perth, WA 6102, Australia; Saishashettyaus@outlook.com (S.S.); Van.H.Nguyen@curtin.edu.au (V.N.); mario.siervo@curtin.edu.au (M.S.)

[4] School of Molecular and Life Sciences, Curtin University, Perth, WA 6102, Australia; R.Coorey@curtin.edu.au (R.C.)

[5] Dementia Centre of Excellence, Enable Institute, Curtin University, Perth, WA 6102, Australia; Blossom.Stephan@curtin.edu.au (B.S.); mario.siervo@curtin.edu.au (M.S.)

[6] Curtin Medical Research Institute (CMRI), Curtin University, Perth, WA 6102, Australia; mario.siervo@curtin.edu.au (M.S.)

# shared last authorship

*Corresponding author

Professor Sirichai Adisakwattana and Professor Mario Siervo

Quality assessment (Risk of bias)

Supplementary Table S1 Risk of bias of observational studies included in the systematic review and meta-analysis using the ROBINS-E assessment tool.

Study	Risk of bias domains							
	D1	D2	D3	D4	D5	D6	D7	Overall
Joshipura et al., 2001								
Bhupathiraju et al., 2013								
Zhang et al., 2011								
Ascherio et al., 1996								
Kim et al., 2018								
Wang et al., 2011								
Li et al., 2022								
Wurtz et al., 2021								
Blekkenhorst et al., 2017								
Blekkenhorst et al., 2018								
Cornelis et al., 2007								
Borgi et al., 2016								
Bendinelli et al., 2011								

Judgement	Code
Low risk of bias	
Some concerns	
High risk of bias	
Very high risk of bias	

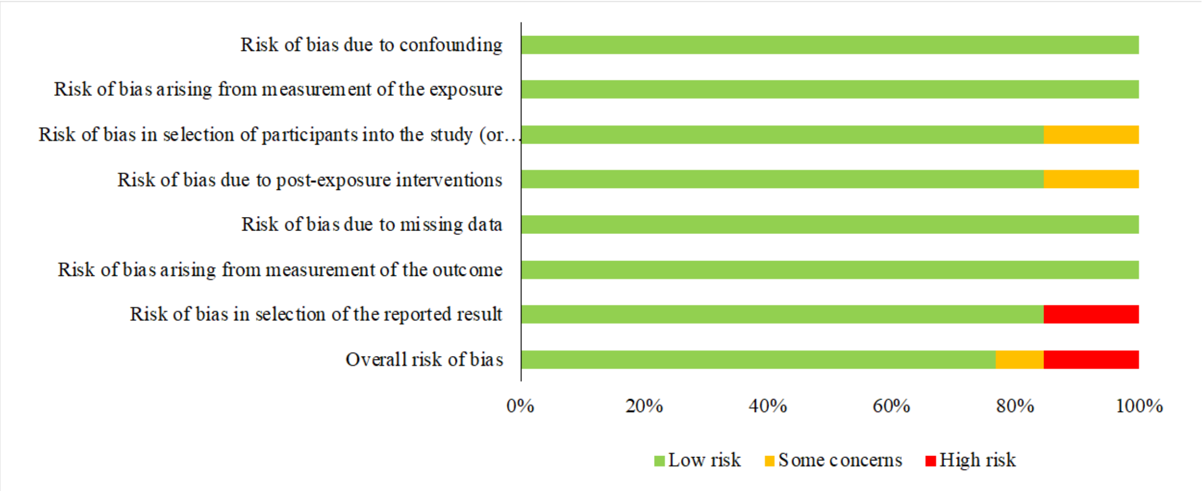

Supplementary Table S2 Risk of bias of randomized controlled trials included in the systematic review and meta-analysis using the RoB2 assessment tool.

Study	Study design	Randomization process	Bias arising from period and carryover effects	Deviations from intended interventions	Mising outcome data	Measurement of the outcome	Selection of the reported result	Overall
Liu et al, 2013	Cross-over	Low	Low	Low	Low	Low	Low	Low
Bondonno et al, 2014	Cross-over	Low	Low	Low	Low	Low	Low	Low
Bondonno et al, 2012	Cross-over	Low	Low	Low	Low	Low	Low	Low
Connolly et al, 2024	Cross-over	Low	Low	Low	Low	Low	Low	Low
Langston-Cox et al, 2021	Cross-over	High	Low	Low	Low	Low	Some concerns	High
Christiansen et al, 2010	Parallel	Low	Low	Low	Low	Low	Low	Low
Sundqvist et al, 2020	Parallel	Low	Low	Low	Low	Low	Low	Low

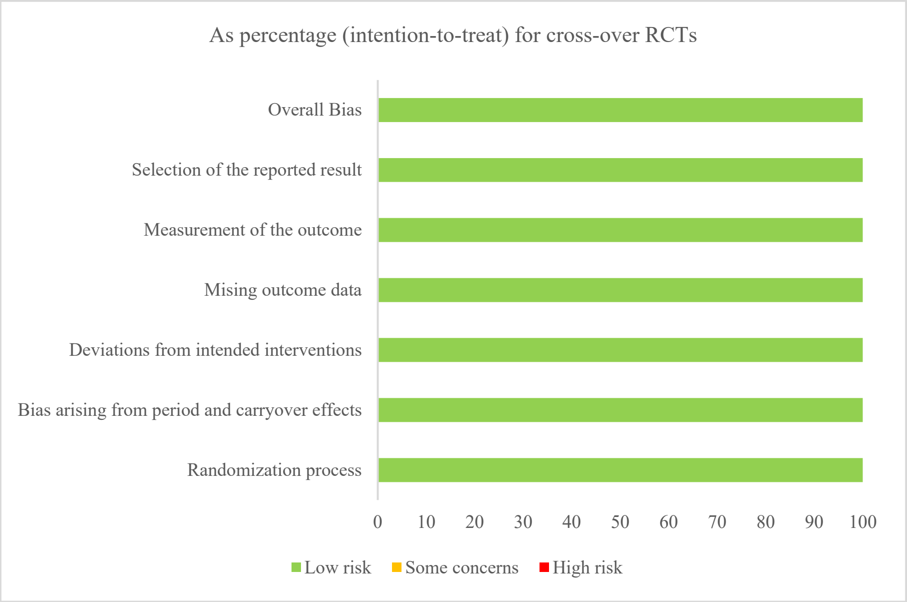

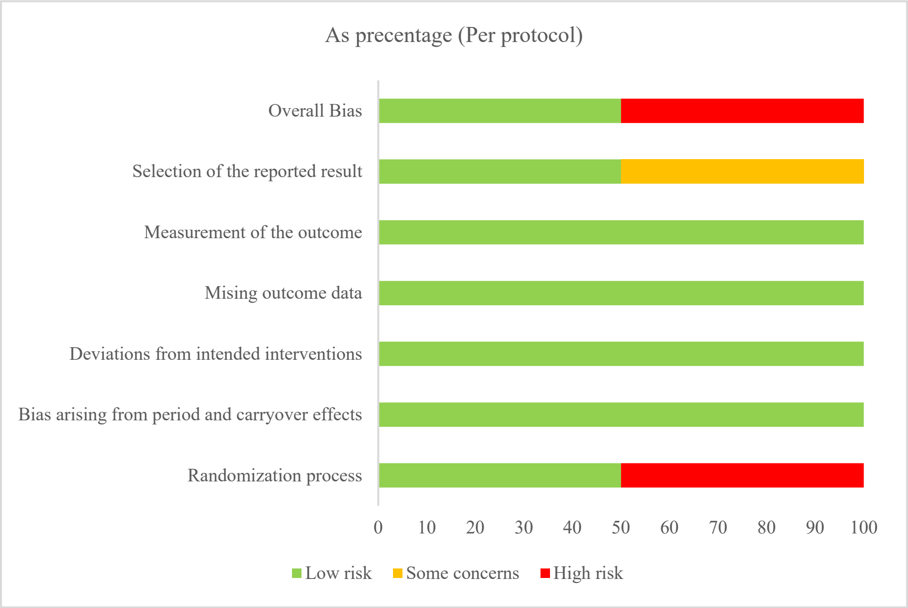

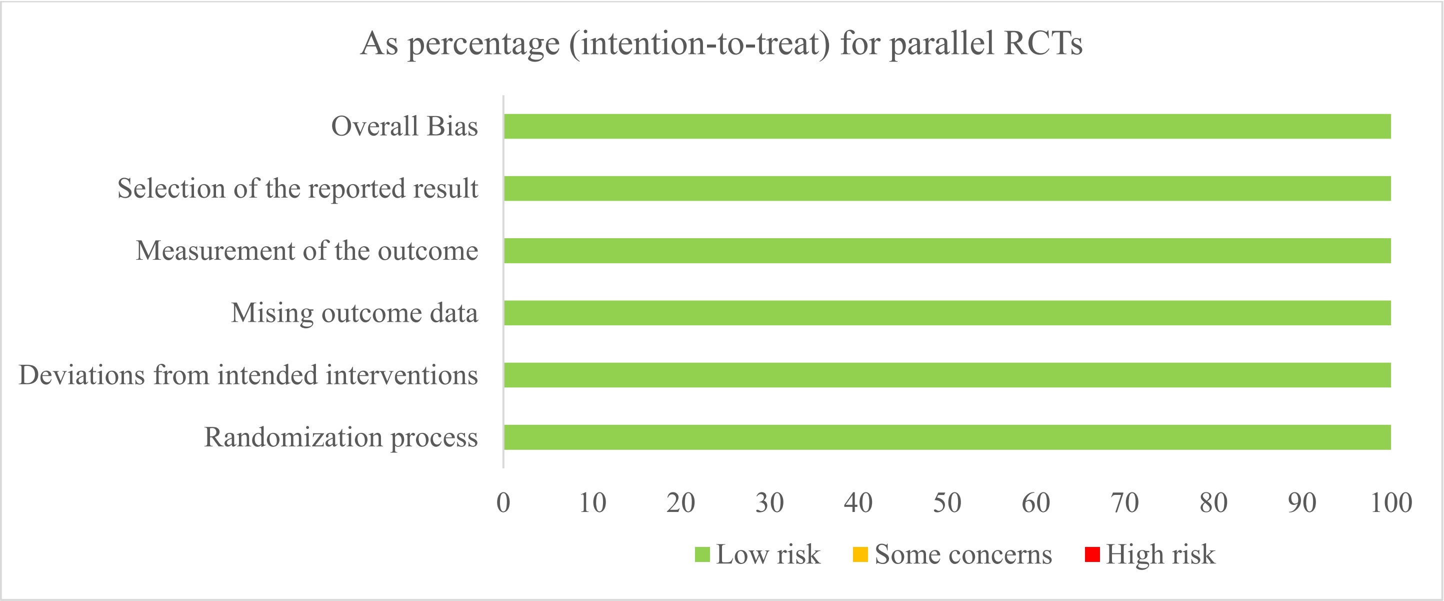
As percentage (intention-to-treat) for parallel RCTs
Overall Bias
Selection of the reported result
Measurement of the outcome
Mising outcome data
Deviations from intended interventions
Randomization process
0
10
20
30
40
50
60
70
80
90
100
Low risk
Some concerns
High risk

Supplementary Table S3

GRADE Analysis

Higher GLV consumption compared to Lower GLV consumption in Improving CVDs health outcome

Patient or population: Improving CVDs health outcome
Setting: Observational studies
Intervention: Higher GLV consumption
Comparison: Lower GLV consumption

Outcomes	Anticipated absolute effects* (95% CI)		Relative effect (95% CI)	№ of participants (studies)	Certainty of the evidence (GRADE)	Comments
	**Risk with Lower GLV consumption**	**Risk with Higher GLV consumption**				
Cardiovascular risks (CVD risk)	4 per 100	**3 per 100** (3 to 4)	**RR 0.89** (0.82 to 0.97)	515729 (13 observational studies)	⊕⊕◯◯ Low[a,b]	The evidence suggests that higher GLV consumption results in a large reduction in cardiovascular risks.
Cardiovascular Mortality (CVD mortality)	14 per 100	**11 per 100** (9 to 14)	**RR 0.79** (0.62 to 0.97)	7624 (13 observational studies)	⊕⊕⊕◯ Moderate[a]	Higher GLV consumption probably reduces the risk of cardiovascular mortality.
Hypertension risk (HT risk)	28 per 100	**28 per 100** (27 to 29)	**RR 1.01** (0.96 to 1.05)	363700 (10 observational studies)	⊕⊕⊕◯ Moderate[a]	Higher GLV consumption likely results in little to no difference in hypertension risk.
Change in vascular function		MD **0.37 fewer** (1.9 fewer to 1.16 more)	-	(4 RCTs)	⊕⊕⊕◯ Moderate[c]	Higher GLV consumption probably does not reduce change in vascular function.
Vascular function assessed with: Pulse wave velocity (PWV)	The mean vascular function was **7.8** m/s	MD **0.16 m/s lower** (0.73 lower to 0.41 higher)	-	(2 RCTs)	⊕⊕⊕◯ Moderate[c]	Higher GLV consumption likely does not reduce vascular function.
Vascular function assessed with: Flow-mediated dilation	The mean vascular function was **4.1** %	MD **0.14 % fewer** (4.49 fewer to 4.21 more)	-	(2 RCTs)	⊕⊕⊕◯ Moderate[c]	Higher GLV consumption likely does not reduce vascular function.
Change in systolic blood pressure (SBP change)	The mean change in systolic blood pressure was **129.2** mmHg	MD **1.42 mmHg lower** (3.43 lower to 0.59 higher)	-	(7 RCTs)	⊕⊕◯◯ Low[c,d]	Higher GLV consumption may result in little to no difference in change in systolic blood pressure.
Change in diastolic blood pressure (DBP change)	The mean change in diastolic blood pressure was **79.6** mmHg	MD **0.37 mmHg higher** (0.86 lower to 1.6 higher)	-	(7 RCTs)	⊕⊕◯◯ Low[c,d]	Higher GLV consumption may not increase change in diastolic blood pressure.

***The risk in the intervention group** (and its 95% confidence interval) is based on the assumed risk in the comparison group and the **relative effect** of the intervention (and its 95% CI).

CI: confidence interval; **MD:** mean difference; **RR:** risk ratio

GRADE Working Group grades of evidence
High certainty: we are very confident that the true effect lies close to that of the estimate of the effect.
Moderate certainty: we are moderately confident in the effect estimate: the true effect is likely to be close to the estimate of the effect, but there is a possibility that it is substantially different.
Low certainty: our confidence in the effect estimate is limited: the true effect may be substantially different from the estimate of the effect.
Very low certainty: we have very little confidence in the effect estimate: the true effect is likely to be substantially different from the estimate of effect.

Funnel plot

Supplementary Figure S1. Funnel plot indicating the presence of publication bias in cardiovascular disease incidence.

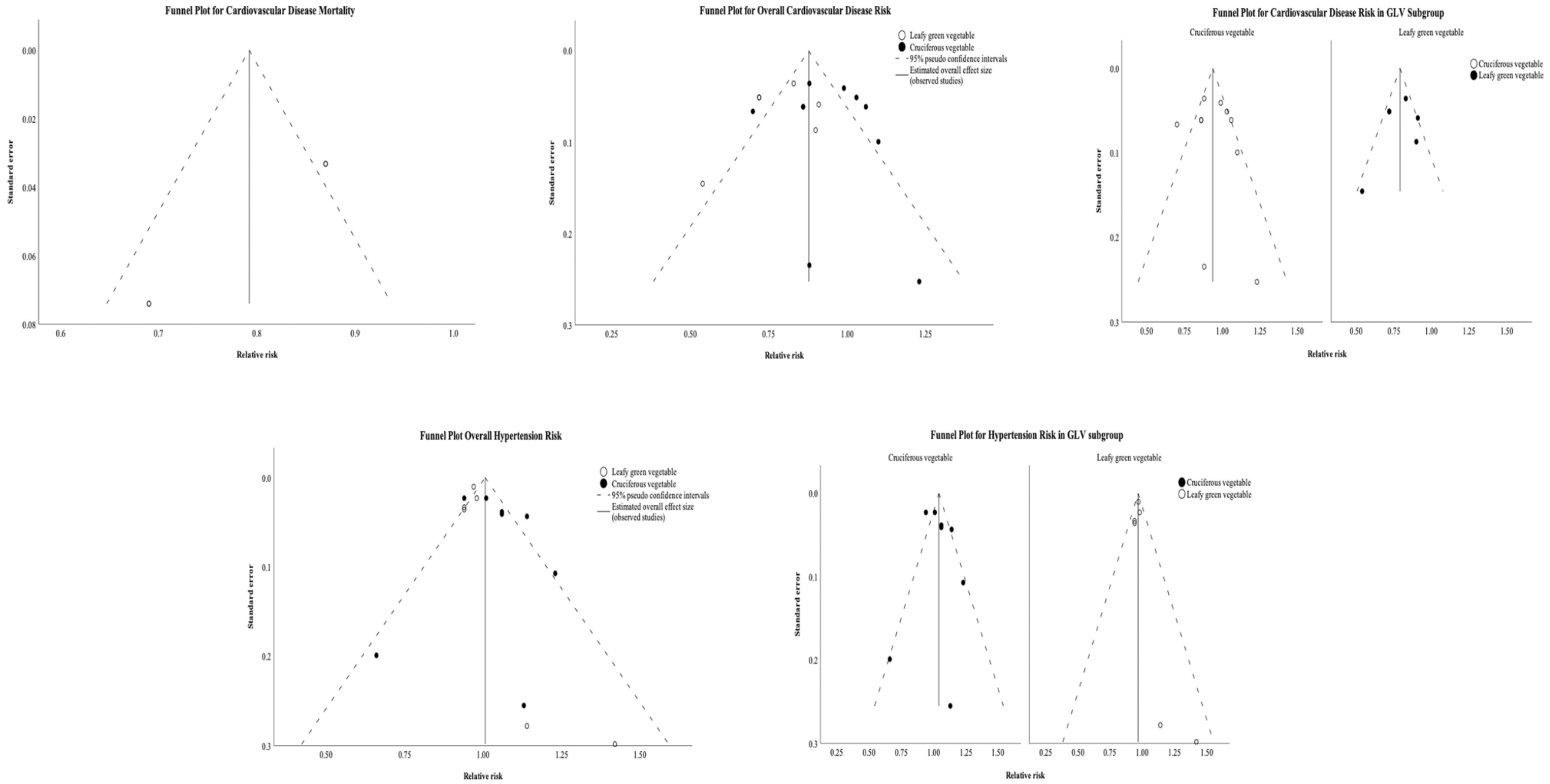

Supplementary Figure S2. Funnel plot indicating the presence of publication bias in vascular function and blood pressure change.

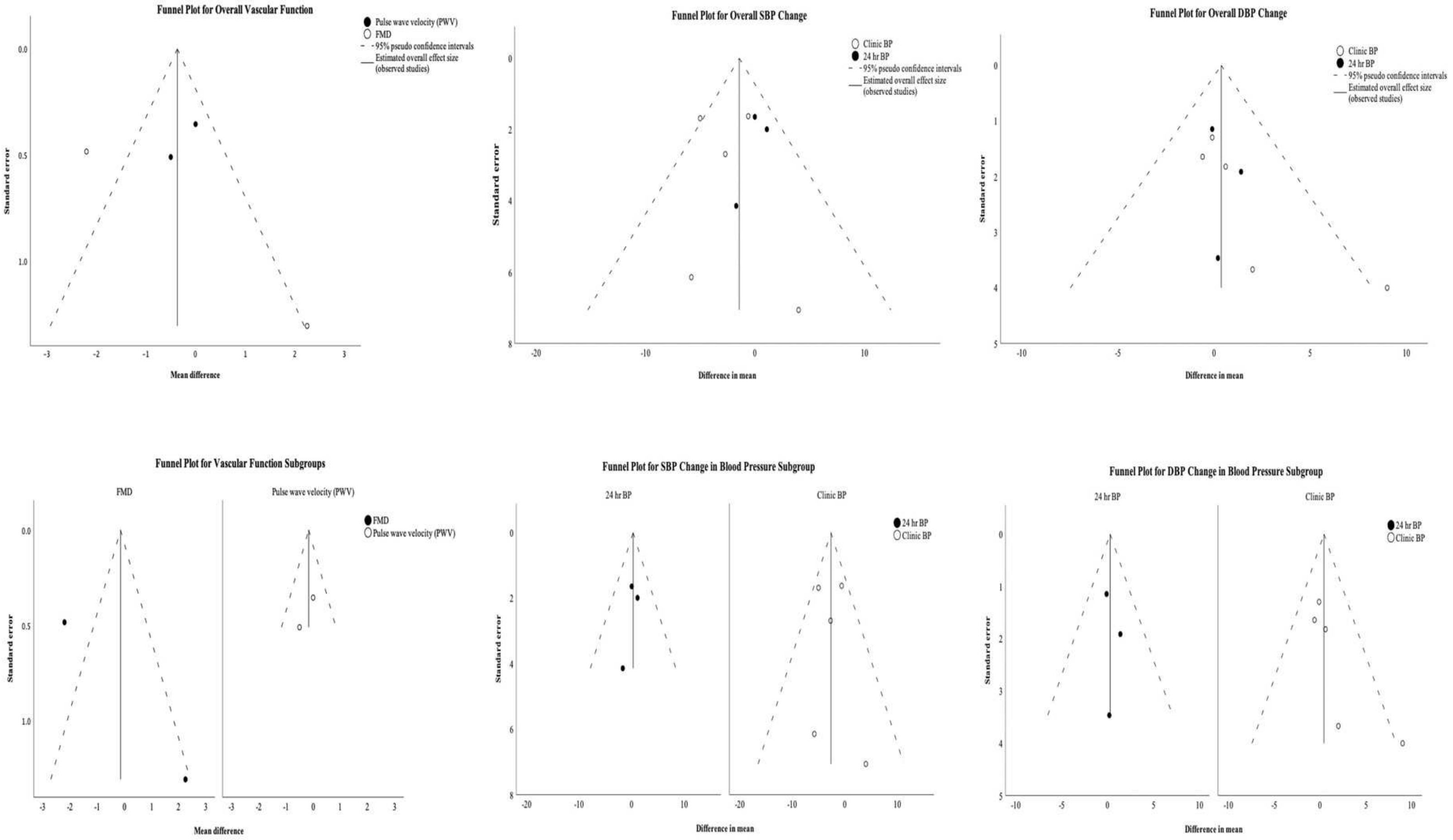

Supplementary Table S4 The Egger’s regression test for publication bias.

Egger's test	Intercept (95% CI)	SE of intercept	*P*-value
CVD risk (RR)	0.88 (0.72, 1.04)	0.07	<0.001
Hypertension risk (RR)	0.98 (0.92, 1.04)	0.03	<0.001
Vascular function (MD)	-2.25 (-8.66, 4.16)	1.49	0.27
SBP (MD)	-1.43 (-6.94, 4.09)	2.25	0.55
DBP (MD)	-2.12 (-6.07, 1.82)	1.61	0.24

Search strategy

Search algorithm (Ovid)

Green leafy vegetables or leafy vegetables or vegetable or cruciferous or kale or spinach or mustard green or collard green or basella or broccoli or cabbage or cauliflower or cardiovascular diseases or coronary heart disease or arterial stiffness or hypertension or platelet aggregation or endothelial function or blood pressure or atherosclerosis {Including Limited Related Terms}

Search term used

Leafy, vegetables, vegetable, cruciferous, kale, spinach, mustard, basella, broccoli, cabbage, cauliflower, hypertension, endothelial, atherosclerosis, collard green, cardiovascular diseases, coronary heart disease, arterial stiffness, platelet aggregation, blood pressure

Database	Search strategy	Results
All journals@Ovid	Green leafy vegetables or leafy vegetables or vegetable or cruciferous or kale or spinach or mustard green or collard green or basella or broccoli or cabbage or cauliflower or cardiovascular diseases or coronary heart disease or arterial stiffness or hypertension or platelet aggregation or endothelial function or blood pressure or atherosclerosis {Including Limited Related Terms}	231

	Search term used:<br><br>Leafy, vegetables, vegetable, cruciferous, kale, spinach, mustard, basella, broccoli, cabbage, cauliflower, hypertension, endothelial, atherosclerosis, collard green, cardiovascular diseases, coronary heart disease, arterial stiffness, platelet aggregation, blood pressure	
Embase	1. 'leaf vegetable'/exp OR 'vegetable'/exp OR 'brassicaceae'/exp<br><br>2. (green leafy vegetable* OR leafy vegetable* OR vegetable* OR cruciferous OR kale OR spinach<br>OR mustard green* OR collard green* OR basella OR broccoli OR cabbage OR cauliflower).tw.<br><br>3. 1 OR 2<br><br>4. 'cardiovascular disease'/exp OR 'hypertension'/exp OR 'coronary heart disease'/exp OR 'atherosclerosis'/exp OR 'arterial stiffness'/exp<br><br>5. (cardiovascular disease* OR coronary heart disease* OR arterial stiffness OR hypertension	1992

	OR platelet aggregation OR endothelial function OR blood pressure OR atherosclerosis).tw.<br>6. 4 OR 5<br>7. 3 AND 6<br><br>Apply Limits:<br>- Human<br>- English<br>- Adult (18+)<br>- Full text	
MEDLINE (Ovid)	1. (green leafy vegetables or leafy vegetables or vegetable* or cruciferous or kale or spinach<br>or mustard green* or collard green* or basella or broccoli or cabbage or cauliflower).tw.<br>2. Vegetables/ or Brassicaceae/<br>3. 1 OR 2<br>4. Cardiovascular Diseases/ or Hypertension/ or Coronary Disease/ or Atherosclerosis/	1756

	or Blood Pressure/<br>5. (cardiovascular disease* or arterial stiffness or hypertension or coronary heart disease* or platelet aggregation or endothelial function or blood pressure or atherosclerosis).tw.<br>6. 4 OR 5<br>7. 3 AND 6<br><br>Apply Limits:<br>- Limit 7 to (Humans)<br>- Limit 7 to (English language)<br>- Limit 7 to (All adults 18+ years)<br>- Limit 7 to (Full text)	
APAsyncInfo	1. (green leafy vegetable* OR leafy vegetable* OR vegetable* OR cruciferous OR kale OR spinach<br>OR mustard green* OR collard green* OR basella OR broccoli OR cabbage OR cauliflower).tw.	243

	2. (cardiovascular disease* OR coronary heart disease* OR arterial stiffness OR hypertension<br>OR endothelial function OR blood pressure OR atherosclerosis OR platelet aggregation).tw.<br>3. 1 AND 2<br><br>Apply Limits:<br>- Human<br>- English<br>- Age Group: Adulthood (18+)<br>- Full text (if available)	
**Pubmed**	(Green Leafy Vegetables[tiab] OR Leafy Vegetables[tiab] OR Vegetables[Mesh] OR vegetable*[tiab]<br>OR cruciferous[tiab] OR cruciferae[Mesh] OR kale[tiab] OR spinach[tiab] OR mustard green*[tiab]	1

OR collard green*[tiab] OR basella[tiab] OR broccoli[tiab] OR cabbage[tiab] OR cauliflower[tiab])

AND

(Cardiovascular Diseases[Mesh] OR cardiovascular disease*[tiab] OR coronary heart disease*[tiab]

OR arterial stiffness[tiab] OR hypertension[Mesh] OR hypertension[tiab] OR platelet aggregation[tiab]

OR endothelial function[tiab] OR blood pressure[Mesh] OR blood pressure[tiab]

OR atherosclerosis[Mesh] OR atherosclerosis[tiab])
